# Supplementary figures and images for: Evaluation of Established Methods for DNA Extraction and Primer Pairs Targeting 16S rRNA Gene for Bacterial Microbiota Profiling of Olive Xylem Sap
Source: Front Plant Sci. 2021 Mar 12;12:640829. doi: 10.3389/fpls.2021.640829 (PMC7994608; doi:10.3389/fpls.2021.640829)

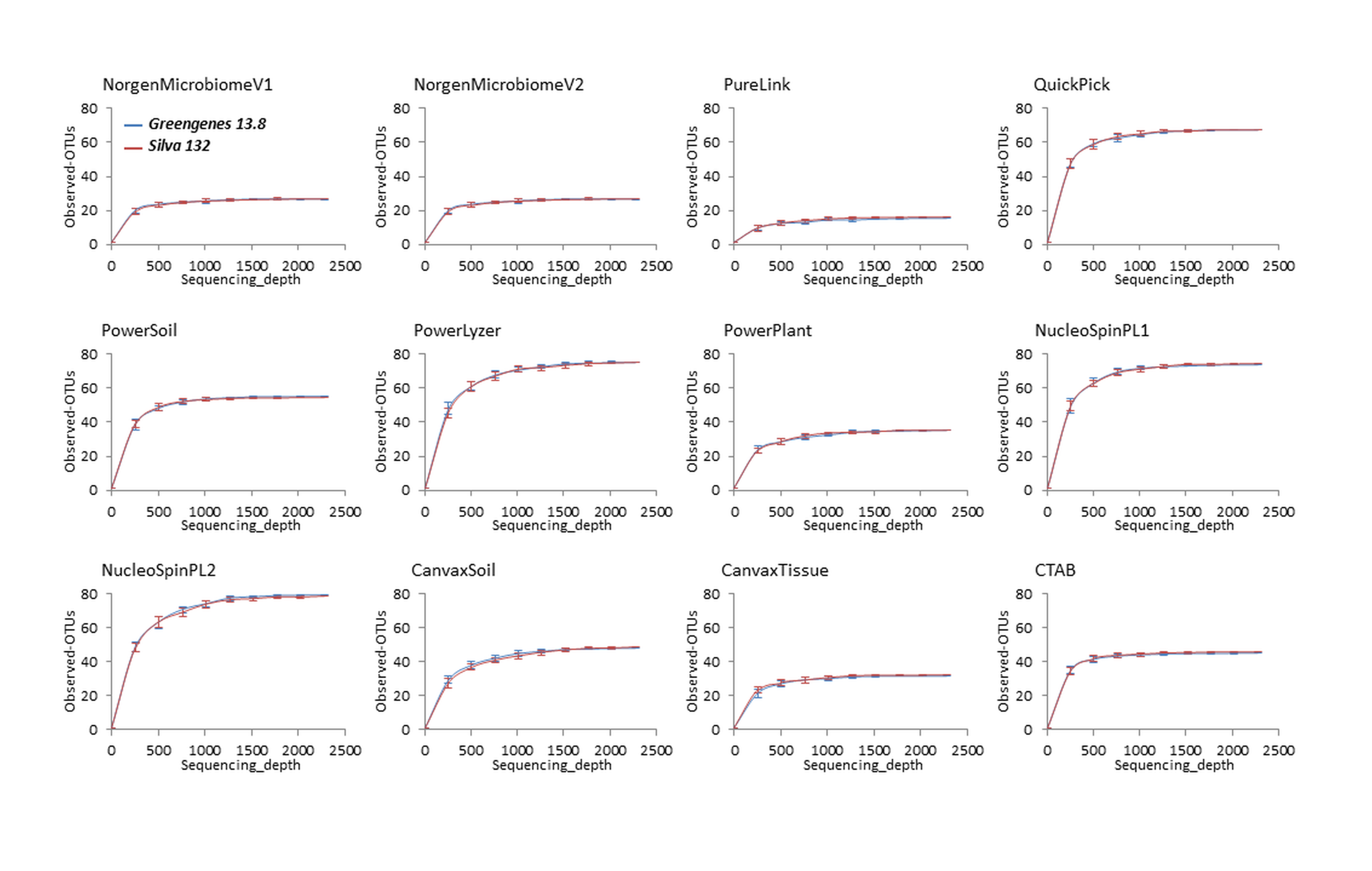

Supplement: Supplementary Figure 1 — Richness rarefaction curves at OTU taxonomic level obtained when using different DNA extraction kits and after taxonomic assignments with the Greengenes_13-8 and Silva_132 databases. Error bars show the standard deviation. [file Image_1.TIF]
