# Supplementary material for: Evaluation of Established Methods for DNA Extraction and Primer Pairs Targeting 16S rRNA Gene for Bacterial Microbiota Profiling of Olive Xylem Sap
Source: Front Plant Sci. 2021 Mar 12;12:640829. doi: 10.3389/fpls.2021.640829 (PMC7994608; doi:10.3389/fpls.2021.640829)
Supplement: Supplementary Figure 2 — Prevalence Venn diagram showing the unique and shared bacterial taxa at phylum, class, order, family, and genera level using the four clustered DNA extraction kits obtained shown in Figure 2. For each taxa, the venn diagram is shown using the Greengenes 13-8 and Silva_132 databases. Tables show the bacterial taxonomy interaction within each reference database. [file Image_2.PDF]

Phylum level

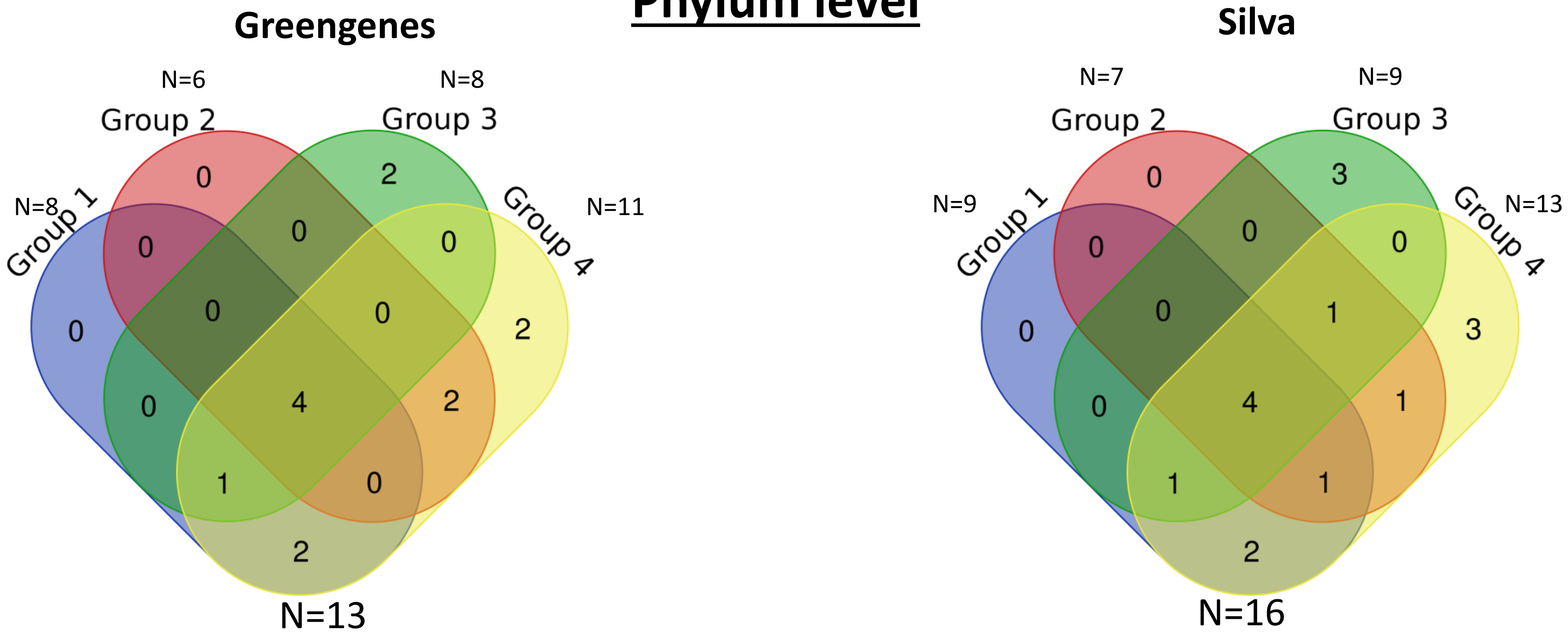

DNA extraction kits	Total	Phylum (Greengenes)	Phylum (Silva)
Group 1 Group 2 Group 3 Group 4	5	Actinobacteria, Proteobacteria, Bacteroidetes, Firmicutes	Bacteroidetes, Actinobacteria, Proteobacteria, Firmicutes
Group 1 Group 2 Group 4	0/1		D_0_Bacteria
Group 1 Group 3 Group 4	1	Acidobacteria	Acidobacteria
Group 2 Group 3 Group 4	0/1		Cyanobacteria
Group 1 Group 4	2	[Thermi], Planctomycetes	Planctomycetes, Deinococcus-Thermus
Group 2 Group 4	1	Fusobacteria	Fusobacteria
Group 3	2/3	Chloroflexi, Nitrospirae	Nitrospirae, Fibrobacteres, Chloroflexi
Group 4	2/3	Verrucomicrobia, Gemmatimonadetes	Saccharibacteria, Gemmatimonadetes, Verrucomicrobia

Class level

Greengenes

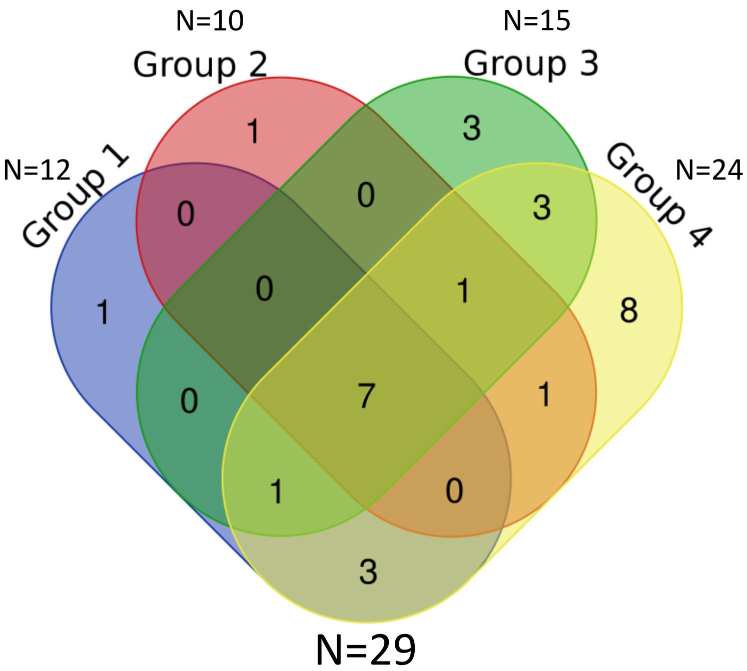

Silva

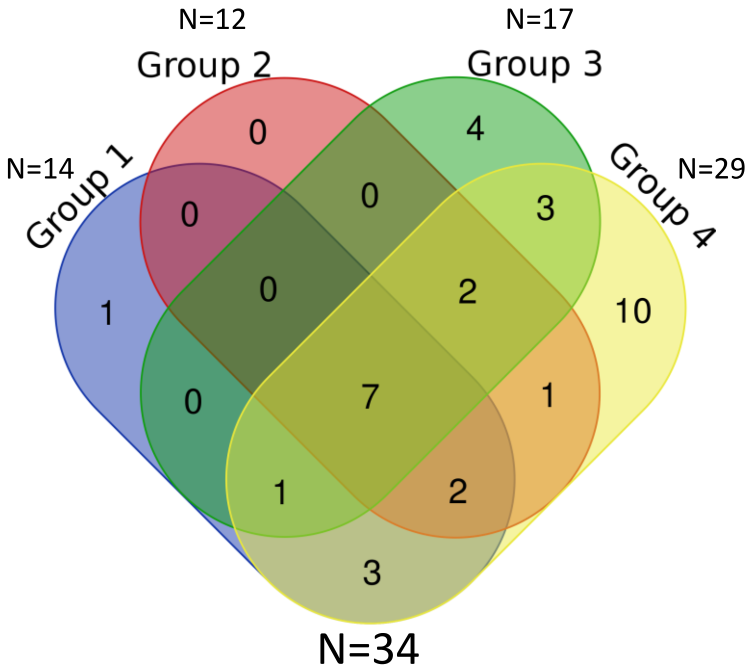

DNA extraction kits	Total	Class (Greengenes)	Class (Silva)
Group 1 Group 2 Group 3 Group 4	7	Alphaproteobacteria, [Saprospirae], Betaproteobacteria, Actinobacteria, Clostridia, Bacilli, Gammaproteobacteria	Alphaproteobacteria, Sphingobacteriia, Bacilli, Gammaproteobacteria, Actinobacteria, Betaproteobacteria, Clostridia
Group 1 Group 2 Group 4	0/2		Negativicutes, D_0_Bacteria
Group 1 Group 3 Group 4	1	Deltaproteobacteria	Deltaproteobacteria
Group 2 Group 3 Group 4	1/2	Bacteroidia	Cyanobacteria, Bacteroidia
Group 1 Group 4	3	Deinococci, Solibacteres, Planctomycetia	Deinococci, Solibacteres, Planctomycetacia
Group 2 Group 4	1	Fusobacteriia	Fusobacteriia
Group 3 Group 4	3	Acidimicrobiia, Cytophagia, Flavobacteriia	Flavobacteriia, Cytophagia, Acidimicrobiia
Group 1	1	DA052	Subgroup 2
Group 2	1/0	Synechococcophycideae	
Group 3	3/4	Nitrospira, Anaerolineae, Holophagae	Holophagae, Nitrospira, Fibrobacteria, Anaerolineae
Group 4	8/10	Gemm-3, Verrucomicrobiae, Erysipelotrichi, Thermoleophilia, [Spartobacteria], OM190, Oscillatoriophycideae, [Rhodothermi]	Bacteroidetes Incertae Sedis, OM190, Verrucomicrobiae D_0_Bacteria;Saccharibacteria, Longimicrobia; Spartobacteria; Gemmatimonadetes; Thermoleophilia, Erysipelotrichia, D_0_Bacteria;Ambiguous_taxa

Order level

Greengenes

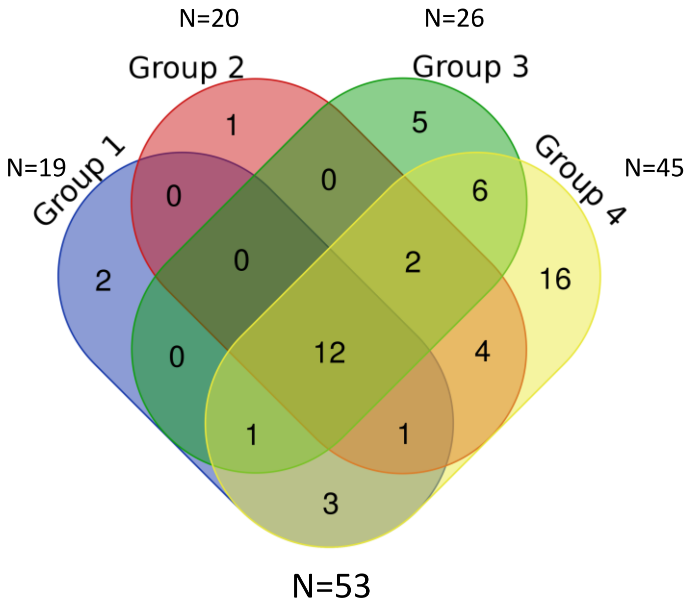

Silva

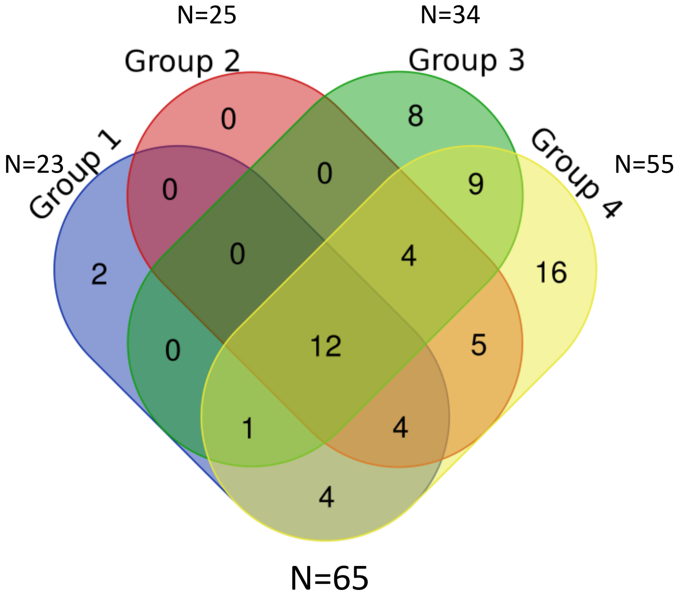

DNA extraction kits	Total	Order (Greengenes)	Order (Silva)
Group 1 Group 2 Group 3 Group 4	12	Bacillales, Pseudomonadales, Sphingomonadales, Xanthomonadales, [Saprospirales], Alteromonadales, Rhizobiales, Clostridiales, Actinomycetales, Gemellales, Burkholderiales, Lactobacillales	Rhizobiales, Xanthomonadales, Propionibacteriales, Lactobacillales, Burkholderiales, Bacillales, Pseudomonadales, Corynebacteriales, Micrococcales, Sphingobacteriales, Sphingomonadales, Clostridiales
Group 1 Group 2 Group 4	1/4	Enterobacteriales	Selenomonadales, Chromatiales, D_0_Bacteria, Enterobacteriales
Group 1 Group 3 Group 4	1	Rhodospirillales	Rhodospirillales
Group 2 Group 3 Group 4	2/4	Bacteroidales, Caulobacterales	Rhodobacterales, Frankiales, Caulobacterales, Bacteroidales
Group 1 Group 4	3/4	Deinococcales, Myxococcales, Solibacterales	Pseudonocardiales, Planctomycetales, Deinococcales, Solibacterales
Group 2 Group 4	4/5	Vibrionales, Neisseriales, Pasteurellales, Fusobacteriales	Neisseriales, SubsectionIII, Pasteurellales, Fusobacteriales, Vibrionales
Group 3 Group 4	6/9	Methylophilales, Rhodocyclales, Rhodobacterales, Flavobacteriales, Cytophagales, Acidimicrobiales	Cytophagales, Kineosporiales, Rickettsiales, SubsectionII, Methylophilales, Actinomycetales, Rhodocyclales, Acidimicrobiales, Flavobacteriales
Group 1	2	Ellin6513, Gemmatales	D_2_Subgroup 2, Oligoflexales
Group 2	1/0	Pseudanabaenales	
Group 3	5/8	Nitrospirales, Holophagales, Bdellovibrionales, c_Betaproteobacteria, SBR1031	Cellvibrionales, Holophagales, Bdellovibrionales, PeM15, Fibrobacterales, Nitrospirales, Nitrosomonadales, Anaerolineales
Group 4	16/16	Ellin329, Chroococcales, Solirubrobacterales, Aeromonadales, Procabacteriales, Oscillatoriales, agg27, Thermales, [Chthoniobacterales], Planctomycetales, Rickettsiales, [Rhodothermales], Verrucomicrobiales, Gemm-3, Erysipelotrichales, Oceanospirillales	Erysipelotrichales, Aeromonadales, Oceanospirillales, Chthoniobacterales, D_2_Betaproteobacteria, Solirubrobacterales, D_0_Bacteria; Ambiguous_taxa, D_2_OM190, Myxococcales, Verrucomicrobiales, Streptosporangiales, Gemmatimonadales, D_1_Saccharibacteria, Longimicrobiales, Order II, Thermales

Family level

Greengenes

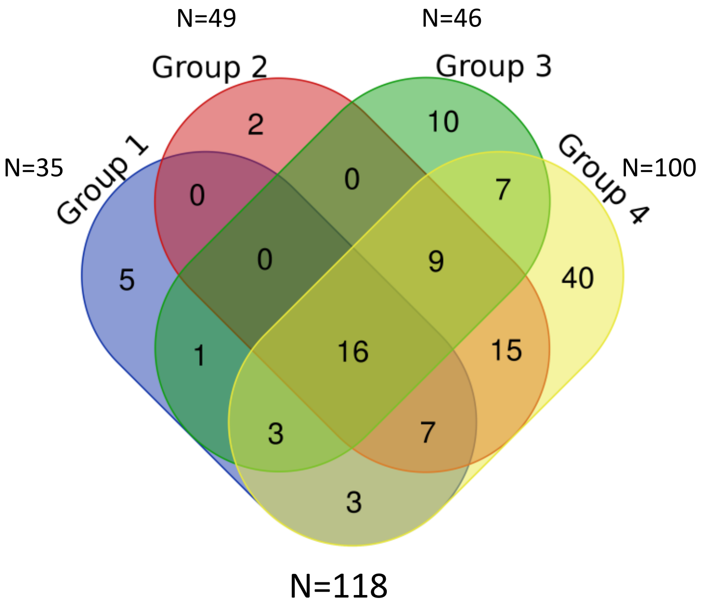

Silva

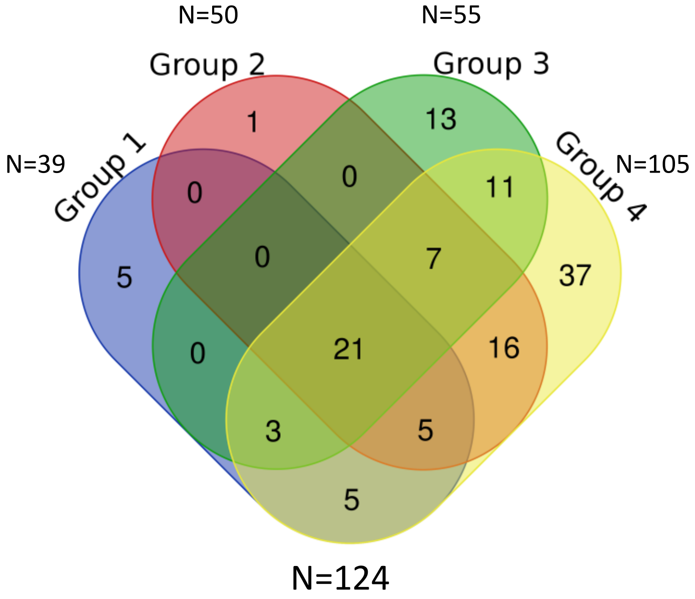

Genus level

Greengenes

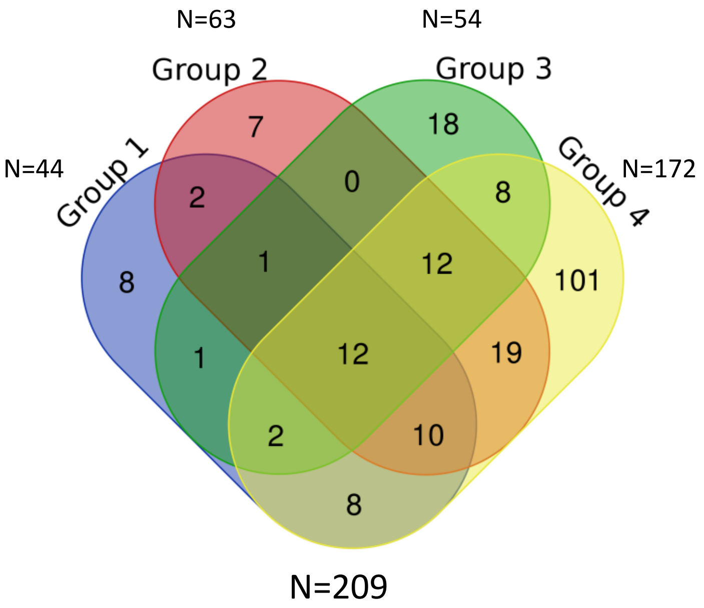

Silva

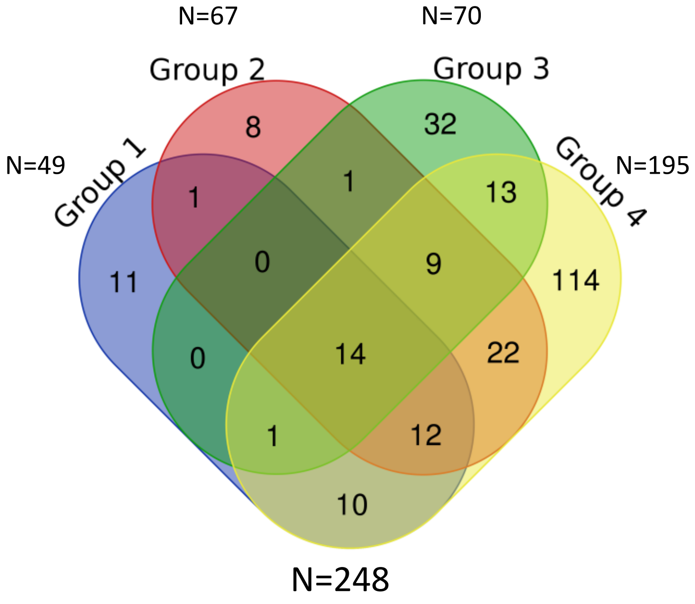

Family level

DNA extraction kits	Total	Family (Greengenes)	Family (Silva)
Group 1 Group 2 Group 3 Group 4	16/21	Corynebacteriaceae, Comamonadaceae, Staphylococcaceae, Chitinophagaceae, Pseudomonadaceae, Lactobacillaceae, Methylobacteriaceae, Streptococcaceae, Rhizobiaceae, Xanthomonadaceae, Gemellaceae, Propionibacteriaceae, [Tissierellaceae], Oxalobacteraceae, Microbacteriaceae, Micrococcaceae	Xanthobacteraceae, Bradyrhizobiaceae, Oxalobacteraceae, Staphylococcaceae, Rhizobiaceae, Comamonadaceae, Family XI (Bacillales), Microbacteriaceae, Micrococcaceae, Corynebacteriaceae, Methylobacteriaceae, Lactobacillaceae, Streptococcaceae, Chitinophagaceae, Family XI (Clostridiales), Pseudomonadaceae, Xanthomonadaceae, Burkholderiaceae, Carnobacteriaceae, Sphingomonadaceae, Propionibacteriaceae
Group 1 Group 2 Group 4	7/5	Burkholderiaceae, Veillonellaceae, [Chromatiaceae], Bradyrhizobiaceae, Enterobacteriaceae, Sphingomonadaceae, o_Lactobacillales	Enterobacteriaceae, Chromatiaceae, Veillonellaceae, D_0_Bacteria, , D_3_Sphingomonadales
Group 1 Group 3 Group 4	3/3	Lachnospiraceae, Alcaligenaceae, Rhodospirillaceae	Lachnospiraceae, Alcaligenaceae, Rhodospirillales Incertae Sedis
Group 2 Group 3 Group 4	9/7	o_Actinomycetales, Nocardioidaceae, ACK-M1, Caulobacteraceae, Hyphomicrobiaceae, [Paraprevotellaceae], o_Rhizobiales, Listeriaceae, Phyllobacteriaceae	Sporichthyaceae, Phyllobacteriaceae, Listeriaceae, Rhodobacteraceae, Prevotellaceae, Nocardioidaceae, Caulobacteraceae
Group 1 Group 3	1/0	Sinobacteraceae	
Group 1 Group 4	3/5	Pseudonocardiaceae, o_Solibacterales, Deinococcaceae	Pseudonocardiaceae, Planctomycetaceae, Deinococcaceae, Solibacteraceae (Subgroup 3), I-10
Group 2 Group 4	15/16	Neisseriaceae, Mycobacteriaceae, Vibrionaceae, Fusobacteriaceae, Paenibacillaceae, Pasteurellaceae, Erythrobacteraceae, Bacillaceae, Moraxellaceae, Ruminococcaceae, Intrasporangiaceae, Porphyromonadaceae, Aerococcaceae, Prevotellaceae, Dermabacteraceae	Intrasporangiaceae, Mycobacteriaceae, Vibrionaceae, FamilyI, Neisseriaceae, Paenibacillaceae, Pasteurellaceae, Dermacoccaceae, Moraxellaceae, Bacillaceae, Porphyromonadaceae, Ruminococcaceae, Aerococcaceae, Erythrobacteraceae, Fusobacteriaceae, Dermabacteraceae
Group 3 Group 4	7/11	Rhodocyclaceae, Cytophagaceae, Methylophilaceae, Nocardiaceae, Actinomycetaceae, Rhodobacteraceae, Flavobacteriaceae	Rhodocyclaceae, Nocardiaceae, Actinomycetaceae, D_3_Frankiales, Kineosporiaceae, Methylophilaceae, Rhizobiales Incertae Sedis, FamilyII, Cytophagaceae, Hyphomicrobiaceae, Flavobacteriaceae
Group 1	5/5	0319-6G20, Methylocystaceae, o_Ellin6513, Xanthobacteraceae, Gemmataceae	0319-6G20, D_2_Subgroup 2, Methylocystaceae, Xanthomonadales Incertae Sedis, Nevskiaceae
Group 2	2/1	Brevibacteriaceae, Pseudanabaenaceae	Brevibacteriaceae
Group 3	10/13	Carnobacteriaceae, Nitrospiraceae, c_Betaproteobacteria, o_Sphingomonadales, A4b, Microthrixaceae, Alteromonadaceae, Holophagaceae, Bdellovibrionaceae, Hyphomonadaceae	Rickettsiales Incertae Sedis, Cryptosporangiaceae, Bdellovibrionaceae, D_3_Xanthomonadales, D_3_PeM15, Anaerolineaceae, Fibrobacteraceae, Hyphomonadaceae, Cellvibrionaceae, Nitrospiraceae, Acidimicrobiales Incertae Sedis, Nitrosomonadaceae, Holophagaceae
Group 4	40/37	Thermoactinomycetaceae, Verrucomicrobiaceae, Sporichthyaceae, Procabacteriaceae, Peptostreptococcaceae, Erysipelotrichaceae, Acetobacteraceae, o_Solirubrobacterales, Haliangiaceae, Gordoniaceae, Rhodothermaceae, Planococcaceae, Xenococcaceae, Leuconostocaceae, o_Rickettsiales, Nocardiopsaceae, o_Myxococcales, Aeromonadaceae, Geodermatophilaceae, Phormidiaceae, Oceanospirillaceae, o_Ellin329, [Exiguobacteraceae], Bacillales, Clostridiaceae, C111, [Chthoniobacteraceae], o_agg27, [Weeksellaceae], Rickettsiaceae, Trueperaceae, [Mogibacteriaceae], Planctomycetaceae, o_Clostridiales, Cellulomonadaceae, c_Gemm-3, Peptococcaceae, Conexibacteraceae, Thermaceae, Williamsiaceae	BIrii41, Clostridiales vadinBB60 group, Trueperaceae, Thermaceae, Rhodothermaceae, Acidimicrobiaceae, Rickettsiaceae, Nocardiopsaceae, Geodermatophilaceae, Sandaracinaceae, Myxococcaceae, D_2_Betaproteobacteria, Chthoniobacteraceae, Thermoactinomycetaceae, Longimicrobiaceae, Family XII, Erysipelotrichaceae, Elev-16S-1332, D_2_OM190, Peptostreptococcaceae, Planococcaceae, D_0_Bacteria; Ambiguous_taxa, Cellulomonadaceae, Acetobacteraceae, Christensenellaceae, Clostridiaceae 1, Peptococcaceae, Haliangiaceae, Gemmatimonadaceae, D_1_Saccharibacteria, Aeromonadaceae, Leuconostocaceae, Verrucomicrobiaceae, Family XIII, Rhodospirillaceae, YNPFFP1, Oceanospirillaceae

Genus level

DNA extraction kits	Total	Genus (Greengenes)	Genus (Silva)
Group 1 Group 2 Group 3 Group 4	12/14	f_Comamonadaceae, Pseudomonas, Sediminibacterium f_Oxalobacteraceae, Streptococcus, Propionibacterium, Microbacterium, Agrobacterium, Lactobacillus, Corynebacterium, Gemellaceae, Staphylococcus	Corynebacterium 1, Microbacterium, Lawsonella, D_4_Comamonadaceae, Rhizobium, Gemella, Propionibacterium, Staphylococcus, Lactobacillus, Streptococcus, Aquabacterium, Granulicatella, Methylobacterium, Pseudomonas
Group 1 Group 2 Group 3	1/0	f_Methylobacteriaceae	
Group 1 Group 2 Group 4	10/12	Stenotrophomonas, Veillonella, f_Enterobacteriaceae, f_Sphingomonadaceae, Rheinheimera, Anaerococcus, Ralstonia, Bradyrhizobiaceae, Afipia, o_Lactobacillales	Massilia, Anaerococcus, Ralstonia, Rheinheimera, D_4_Chitinophagaceae, D_0_Bacteria, Pantoea, Sphingomonas, Bradyrhizobium, Veillonella, D_3_Sphingomonadales, Stenotrophomonas
Group 1 Group 3 Group 4	2/1	f_Xanthomonadaceae, f_Rhodospirillaceae	Massilia
Group 2 Group 3 Group 4	12/9	o_Actinomycetales, f_Nocardioidaceae, f_ACK-M1, Finegoldia, Rhodoplanes, Brochothrix, o_Rhizobiales, f_Phyllobacteriaceae, Mycoplana, [Prevotella], f_Microbacteriaceae, Kocuria	Bosea, Brochothrix, Brevundimonas, hgcI clade, Alloprevotella, Paracoccus, Mesorhizobium, Finegoldia, Kocuria
Group 1 Group 2	2/1	Burkholderia, Bradyrhizobium	Burkholderia-Paraburkholderia
Group 1 Group 3	1/0	f_Sinobacteraceae	
Group 1 Group 4	8/10	Achromobacter, Pseudonocardia, Micrococcus, Delftia,o_Solibacterales, Deinococcus, Novosphingobium, Janthinobacterium	Delftia, Achromobacter, Escherichia-Shigella, Enterobacter, Novosphingobium, Deinococcus, Bryobacter, D_4_I-10, Micrococcus, Pseudonocardia
Group 2 Group 3	0/1		Variibacter
Group 2 Group 4	19/22	Bacillus, Mycobacterium, Neisseria, f_Erythrobacteraceae, Diaphorobacter, Peptoniphilus, f_Aerococcaceae, Paenibacillus, Fusobacterium, Haemophilus, Porphyromonas, Methylobacterium, Prevotella, f_Intrasporangiaceae, Acinetobacter, Aerococcus, Anoxybacillus, Rothia, Photobacterium	D_4_Intrasporangiaceae, D_4_Enterobacteriaceae, D_4_Vibrionaceae, Anoxybacillus, Paenibacillus, Aerococcus, Peptoniphilus, Porphyromonas, Fusobacterium, Porphyrobacter, Rothia, Haemophilus, Abiotrophia, Dermacoccus, Prevotella, Pseudoclavibacter, Diaphorobacter, Acinetobacter, Neisseria, Nocardioides, Mycobacterium, Bacillus
Group 3 Group 4	8/13	Hyphomicrobium, f_Lachnospiraceae, Paracoccus, f_Methylophilaceae, Flavobacterium, Actinomyces, Limnohabitans, Rhodococcus	Actinomyces, D_3_Frankiales, Methylotenera, Rubellimicrobium, D_4_Microbacteriaceae, Limnohabitans, Rhodococcus, Rhodobacter, Marmoricola, Hyphomicrobium, D_4_Xanthomonadaceae, Flavobacterium, D_4_Sphingomonadaceae
Group 1	8/11	f_0319-6G20, Roseburia, o_Ellin6513, Labrys, Gemmata, Actinomycetospora, Nevskia, Methylosinus	D_4_0319-6G20, Labrys, Duganella, Methylosinus, Luteibacter, Gemmata, D_2_Subgroup 2, Nevskia, Actinomycetospora, Roseburia, Acidibacter
Group 2	7/8	Dermabacter, Moraxella, Klebsiella, f_Ruminococcaceae, Brevibacterium, Sphingomonas, Leptolyngbya	D_4_Bradyrhizobiaceae, Leptolyngbya, Brevibacterium, Moraxella, Knoellia, D_4_Dermabacteraceae, Klebsiella, [Eubacterium] coprostanoligenes group
Group 3	18/32	Cellvibrio, Candidatus Rhodoluna, Dechloromonas, Bdellovibrio Methylibium, c_Betaproteobacteria, f_Chitinophagaceae, Candidatus Aquiluna, o_Sphingomonadales, Granulicatella, f_Alcaligenaceae, f_Cytophagaceae, f_A4b, f_Microthrixaceae, Nitrospira, Methylotenera, f_Holophagaceae, f_Hyphomonadaceae	PRD01a011B, D_4_Rickettsiales Incertae Sedis, Undibacterium, Polynucleobacter, Sediminibacterium, Candidatus Aquiluna, Pleurocapsa, Woodsholea, Phreatobacter, Nitrospira, Dechloromonas, D_4_Fibrobacteraceae, Fodinicola, Bdellovibrio, Pseudokineococcus, D_4_Nitrosomonadaceae, Cellvibrio, GKS98 freshwater group, Niastella, D_3_Xanthomonadales, D_3_PeM15, Lachnoclostridium 5, Pseudarcicella, D_4_Anaerolineaceae, Methylophilus, Holophaga, Candidatus Microthrix, D_4_Methylophilaceae, Rhizobacter, Candidatus Planktoluna, OM43 clade, Candidatus Rhodoluna
Group 4	101/114	Rubricoccus, Williamsia, f_Peptostreptococcaceae, f_Neisseriaceae, Faecalibacterium, f_Haliangiaceae, Arthrobacter, Nocardioides f_Pseudomonadaceae, f_Planococcaceae, Flavisolibacter, Exiguobacterium, f_Aeromonadaceae, f_Geodermatophilaceae, Clostridium, f_[Chromatiaceae], Hymenobacter, Parasegitibacter, Saccharopolyspora, o_Ellin329, Ruminococcus, o_Bacillales, Mogibacterium, Virgibacillus, Curtobacterium, f_C111, Selenomonas, Coprococcus, Tepidimonas, f_Propionibacteriaceae, Planomicrobium, Adhaeribacter, Erwinia, Aggregatibacter, o_Clostridiales, Chryseobacterium, Skermanella, c_Gemm-3, Marinobacterium, Blautia, Kaistobacter, Alloiococcus, f_Thermoactinomycetaceae, f_Sporichthyaceae, Chthoniobacter, f_Procabacteriaceae, Vibrio, f_Acetobacteraceae, o_Solirubrobacterales, f_Rhodocyclaceae, Janibacter, Gordonia, Prauseria, Brachybacterium, f_Xenococcaceae, Brevundimonas, Brevibacillus, Capnocytophaga, f_Leuconostocaceae, o_Rickettsiales, Planctomyces, f_Nocardiopsaceae, Marinomonas, Planktothrix, o_Myxococcales, Luteolibacter, Rickettsia, Balneimonas, Microbispora, Truepera, f_Moraxellaceae, Parvimonas, Lautropia, Actinotalea, f_Caulobacteraceae, Enhydrobacter, Agrococcus, f_Clostridiaceae, Megamonas, Geobacillus, Kingella, Peptostreptococcus, Catonella, o_agg27, f_[Weeksellaceae], Pseudoclavibacter, Bulleidia, Meiothermus, Comamonas, Asticcaulis, Leucobacter, Peptococcus, f_Rhodobacteraceae, Aeromicrobium, f_Micrococcaceae, Deefgea, f_Conexibacteraceae, Limnobacter, Gemella, Rhodobacter, Propionicimonas	Capnocytophaga, Arenimonas, D_4_Rhodocyclaceae, Glutamicibacter, Brachybacterium, D_4_BIrii41, Aestuariimicrobium, Atopostipes, Parasegetibacter, Virgibacillus, Johnsonella, Geobacillus, Prevotella 2, Tepidimonas, Clostridium sensu stricto 1, Brevibacillus, Selenomonas 3, Rhizomicrobium, Comamonas, Catonella, Arthrobacter, Frondihabitans, D_4_Neisseriaceae, Propioniciclava, Prevotella 7, Adhaeribacter, Pseudarthrobacter, CL500-29 marine group, Peptococcus, Vibrio, D_4_Pasteurellaceae, Luteolibacter, Skermanella, Falsirhodobacter, Faecalibacterium, Agrococcus, Marinobacterium, D_2_OM190, D_4_Planococcaceae, Aggregatibacter, Aeromonas, Shinella, Chroococcidiopsis, Paraclostridium, Dolosigranulum, Bergeyella, D_1_Saccharibacteria, [Agitococcus] lubricus group, Paeniglutamicibacter, Aeromicrobium, Craurococcus, Nocardiopsis, Meiothermus, Hymenobacter, Exiguobacterium, Williamsia, Asticcacaulis, Serratia, Coprococcus 2, Marinomonas, Pectobacterium, Lautropia, [Eubacterium] yurii group, D_4_Xanthobacteraceae, Rickettsia, Schlesneria, Solobacterium, D_4_Clostridiales vadinBB60 group, Corynebacterium, Planktothrix, Kingella, Acidovorax, Gordonia, Parvimonas, D_4_Sandaracinaceae, Blautia, D_4_Myxococcaceae, D_2_Betaproteobacteria, Megamonas, Saccharopolyspora, Leucobacter, D_4_Oxalobacteraceae, Microvirga, D_4_Longimicrobiaceae, D_0_Bacteria;Ambiguous_taxa, Intestinibacter, D_4_Elev-16S-1332, Deefgea, Planomicrobium, Mogibacterium, Weissella, D_4_Cellulomonadaceae, Curtobacterium, Prevotella 9, D_4_Gemmatimonadaceae, Limnobacter, D_4_Ruminococcaceae, Chryseobacterium, Blastococcus, Haliangium, Enhydrobacter, Rubrivirga, Laceyella, Erwinia, Christensenellaceae R-7 group, Truepera, Quadrisphaera, Chthoniobacter, Oceanirhabdus, Zimmermannella, Flavisolibacter, D_4_YNPFFP1, Janibacter, Peptostreptococcus
