# Supplementary material for: Evaluation of Established Methods for DNA Extraction and Primer Pairs Targeting 16S rRNA Gene for Bacterial Microbiota Profiling of Olive Xylem Sap
Source: Front Plant Sci. 2021 Mar 12;12:640829. doi: 10.3389/fpls.2021.640829 (PMC7994608; doi:10.3389/fpls.2021.640829)
Supplement: Supplementary Figure 3 — Prevalence Venn diagram showing the unique and shared bacterial taxa obtained at phylum, class, order, family, and genera level using the four PCR protocols. Tables show the bacterial taxonomy interaction within each PCR protocol. [file Image_3.PDF]

Phylum level

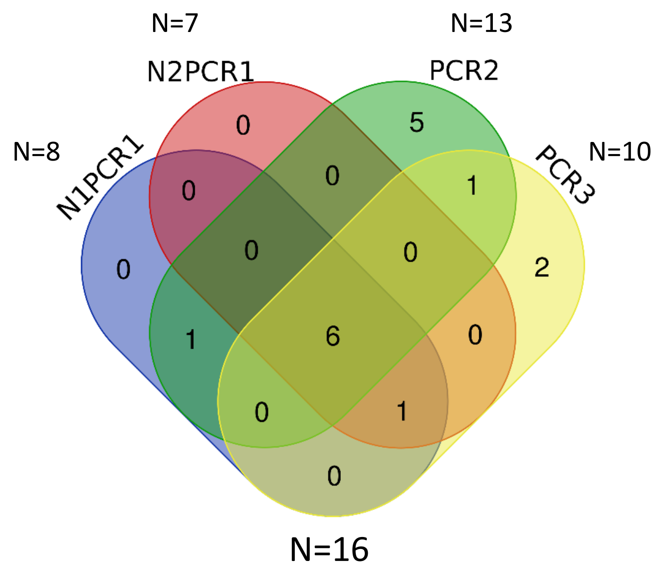

PCRs	Total	Phylum
N1PCR1 N2PCR1 PCR2 PCR3	6	Actinobacteria, Firmicutes, Deinococcus-Thermus, Proteobacteria, Fusobacteria, Gemmatimonadetes
N1PCR1 N2PCR1 PCR3	1	Bacteroidetes
N1PCR1 PCR2	1	Acidobacteria
PCR2 PCR3	1	Chloroflexi
PCR2	5	Dependentiae, Verrucomicrobia, Nitrospirae, Planctomycetes, Cyanobacteria
PCR3	2	Armatimonadetes, Patescibacteria

Class level

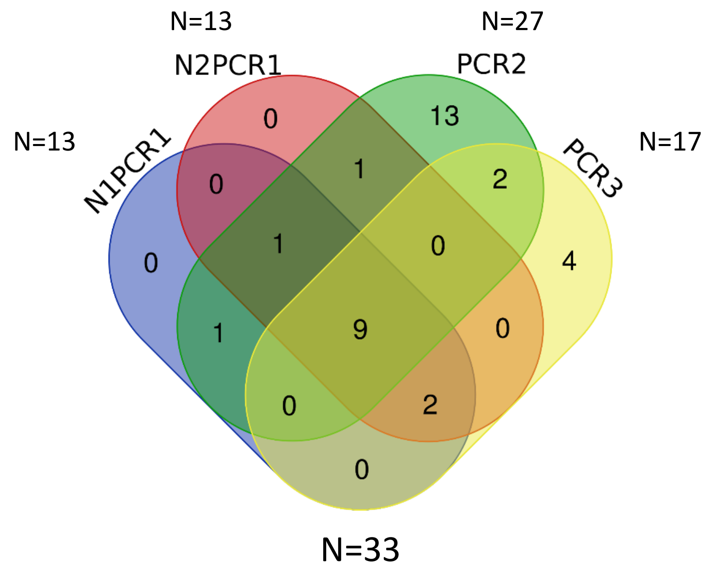

PCRs	Total	Class
N1PCR1 N2PCR1 PCR2 PCR3	9	Alphaproteobacteria, Fusobacteriia, Bacilli, Deinococci, Gammaproteobacteria, Actinobacteria, Acidimicrobiia, Thermoleophilia, Clostridia
N1PCR1 N2PCR1 PCR2	1	Negativicutes
N1PCR1 N2PCR1 PCR3	2	Longimicrobia, Bacteroidia
N1PCR1 PCR2	1	Holophagae
N2PCR1 PCR2	1	Coriobacteriia
PCR2 PCR3	2	Deltaproteobacteria, Gemmatimonadetes
PCR2	13	Gitt-GS-136, Blastocatellia (Subgroup 4), Verrucomicrobiae, Nitrospira, Oxyphotobacteria, Subgroup 6, S0134 terrestrial group, Anaerolineae, Chloroflexia, KD4-96, Babeliae, Erysipelotrichia, Planctomycetacia
PCR3	4	Saccharimonadia, Rubrobacteria, TK10 Armatimonadia

Order level

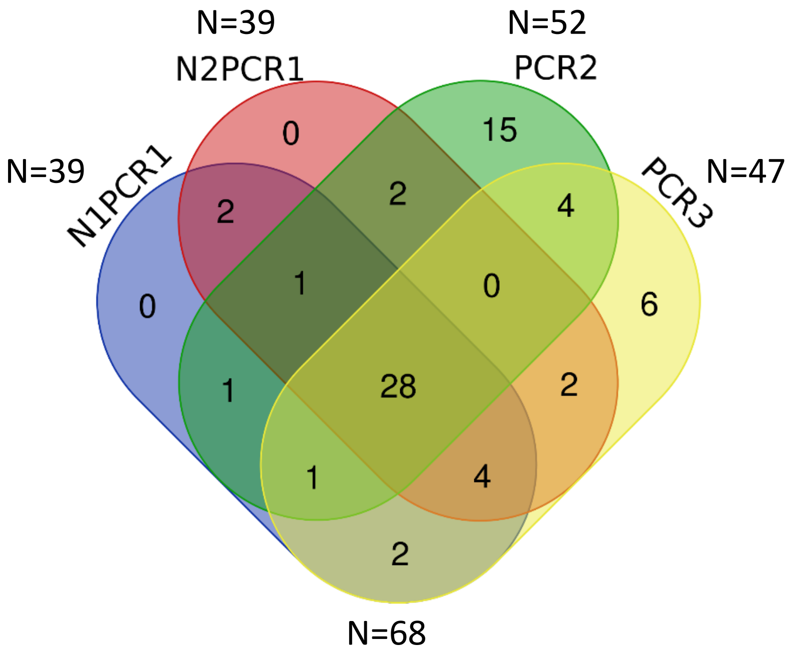

PCRs	Total	Order
N1PCR1 N2PCR1 PCR2 PCR3	28	Rhizobiales, Acetobacterales, Pseudonocardiales, Kineosporiales, Oceanospirillales, Xanthomonadales, Propionibacteriales, Betaproteobacteriales, Rhodobacterales, Pasteurellales, Solirubrobacterales, Caulobacterales, Frankiales, Lactobacillales, Deinococcales, Bacillales, Alteromonadales, Pseudomonadales, Fusobacteriales, Corynebacteriales, Micrococcales, Microtrichales, Actinomycetales, Vibrionales, Sphingomonadales, Enterobacteriales, Clostridiales, Thermales
N1PCR1 N2PCR1 PCR2	1	Selenomonadales
N1PCR1 N2PCR1 PCR3	4	Flavobacteriales, Azospirillales, Longimicrobiales, Bacteroidales
N1PCR1 PCR2 PCR3	1	Streptomycetales
N1PCR1 N2PCR1	2	Sneathiellales, Sphingobacteriales
N1PCR1 PCR2	1	Subgroup 7
N1PCR1 PCR3	2	Cellvibrionales, Micromonosporales
N2PCR1 PCR2	2	Aeromonadales, Coriobacteriales
N2PCR1 PCR3	2	Cytophagales, Chitinophagales
PCR2 PCR3	4	Gaiellales, Myxococcales, Streptosporangiales, Gemmatimonadales
PCR2	15	Erysipelotrichales, Bdellovibrionales, Babeliales, Blastocatellales, Gemmatales, Chthoniobacterales, Salinisphaerales, SJA-15, Nitrospirales Unknown Order, Thermomicrobiales, Bifidobacteriales, Nostocales, Verrucomicrobiales, Opitutales
PCR3	6	Rubrobacterales, Armatimonadales, Saccharimonadales, Gammaproteobacteria Incertae Sedis, Oligoflexales, Desulfovibrionales

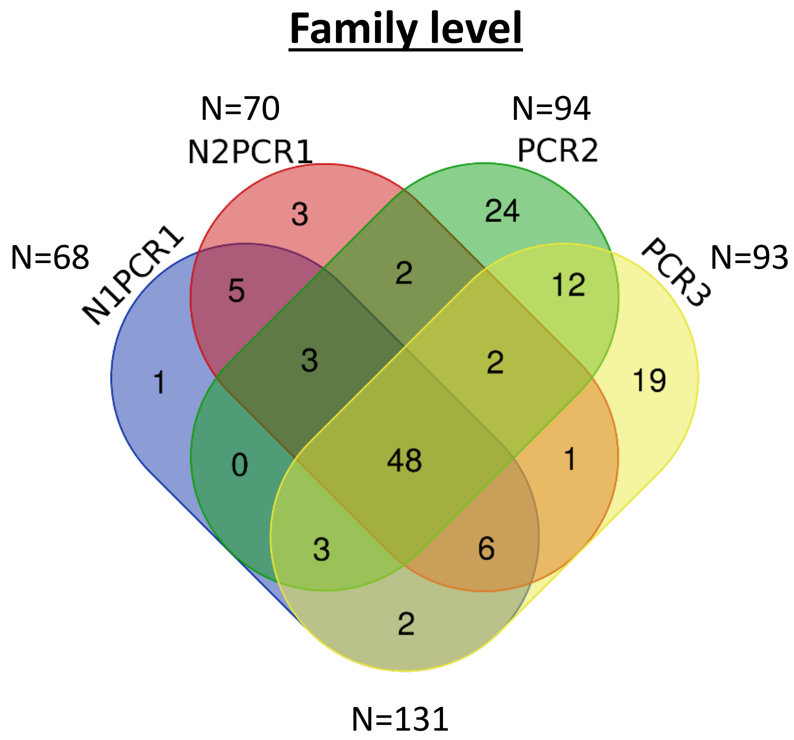
Family level
N=70
N2PCR1
N=94
PCR2
N=68
N1PCR1
PCR3
N=93
N=131
3
24
5
2
12
1
3
2
19
0
48
1
3
6
2

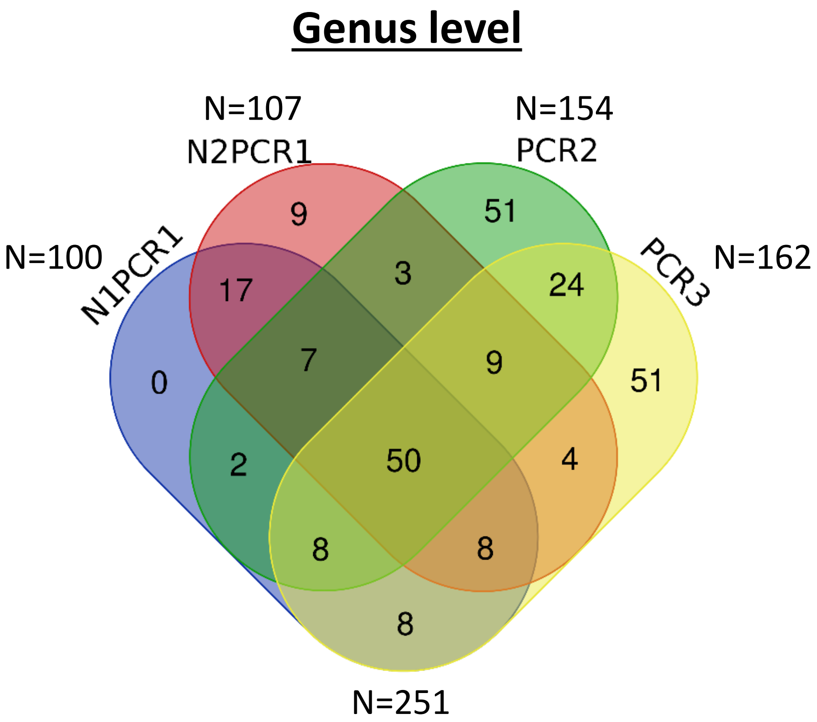
Genus level
N=107
N2PCR1
N=154
PCR2
N=100
N1PCR1
PCR3
N=162
N=251
9
51
17
3
24
0
7
9
51
2
50
4
8
8
8

Family level

PCRs	Total	Family
N1PCR1 N2PCR1 PCR2 PCR3	48	Intrasporangiaceae, Enterobacteriaceae, Vibrionaceae, Iamiaceae, Sanguibacteraceae, Thermaceae, Geodermatophilaceae, Neisseriaceae, Paenibacillaceae, Staphylococcaceae, Rhizobiaceae, Marinomonadaceae, Pasteurellaceae, Dermacoccaceae, Kineosporiaceae, Planococcaceae, Microbacteriaceae, Micrococcaceae, Rhodobacteraceae, Bacillaceae, Corynebacteriaceae, Streptococcaceae, Nocardioidaceae, Pseudomonadaceae, Fusobacteriaceae, Carnobacteriaceae, Brevibacteriaceae, Family, XI, Solirubrobacteraceae, Leptotrichiaceae, Pseudonocardiaceae, Beijerinckiaceae, Xanthobacteraceae, Enterococcaceae, Listeriaceae, Deinococcaceae, Actinomycetaceae, Moraxellaceae, Lachnospiraceae, Cellulomonadaceae, Acetobacteraceae, Ilumatobacteraceae, Lactobacillaceae, Xanthomonadaceae, Burkholderiaceae, Caulobacteraceae, Sphingomonadaceae, Propionibacteriaceae
N1PCR1 N2PCR1 PCR2	3	Promicromonosporaceae, Nocardiaceae, Veillonellaceae
N1PCR1 N2PCR1 PCR3	6	Prevotellaceae, Dermabacteraceae, Weeksellaceae, Longimicrobiaceae, Azospirillaceae, Flavobacteriaceae
N1PCR1 PCR2 PCR3	3	Rhodanobacteraceae, Streptomycetaceae, Dermatophilaceae
N2PCR1 PCR2 PCR3	2	Sporolactobacillaceae, Ruminococcaceae
N1PCR1 N2PCR1	5	Sporichthyaceae, Shewanellaceae, Sneathiellaceae, Porphyromonadaceae, Methylophilaceae
N1PCR1 PCR3	2	Cellvibrionaceae, Micromonosporaceae
N2PCR1 PCR2	2	Aeromonadaceae, Leuconostocaceae
N2PCR1 PCR3	1	Chitinophagaceae
PCR2 PCR3	12	Mycobacteriaceae, 67-14, Clostridiaceae, 1, Aerococcaceae, Unknown, Family, Nocardiopsaceae, Gaiellaceae, Streptosporangiaceae, Alteromonadaceae, Idiomarinaceae, Gemmatimonadaceae, Hyphomicrobiaceae
N1PCR1	1	NS11-12 marine group
N2PCR1	3	Spirosomaceae Sphingobacteriaceae Coriobacteriaceae
PCR2	24	Solimonadaceae, Rhodocyclaceae, Nostocaceae, Bifidobacteriaceae, Blastocatellaceae, Chroococcidiopsaceae, Chthoniobacteraceae, Psychromonadaceae, Thermoactinomycetaceae, Rubritaleaceae, Peptostreptococcaceae, Frankiaceae, Nitrospiraceae, Family, XIII, Opitutaceae, Bdellovibrionaceae, Atopobiaceae, Family, XVII, Myxococcaceae, Family, X, Erysipelotrichaceae, Vermiphilaceae, Gemmataceae, JG30-KF-CM45
PCR3	19	Bacteroidaceae, 0319-6G20, BIrii41, Devosiaceae, Trueperaceae, Dysgonomonadaceae, Hymenobacteraceae, Desulfovibrionaceae, Nitrosomonadaceae, Rubrobacteriaceae, Halomonadaceae, Jonesiaceae, Demequinaceae, Phaselicystidaceae, Microtrichaceae, Saccharimonadaceae, Haliangiaceae, Prolixibacteraceae, Nakamurellaceae

Genus level

PCRs	Total	Genus
N1PCR1 N2PCR1 PCR2 PCR3	50	Schlegelella, Turicella, Listeria, Actinomyces, Psychrobacter, Brevibacillus, Arthrobacter, Brochothrix, Lawsonella, Peptoniphilus, Brevibacterium, Vibrio, Massilia, Enterococcus, Anaerococcus, Ralstonia, Leptotrichia, Fusobacterium, Dolosigranulum, Gemella, Meiothermus, Rothia, Haemophilus, Novosphingobium, Pectobacterium, Marinomonas, Paracoccus, Sphingomonas, Dermacoccus, Cutibacterium, Bradyrhizobium, Cellulomonas, Iamia, Staphylococcus, Corynebacterium, Deinococcus, Lactobacillus, Microvirga, Roseomonas, Nocardioides, Streptococcus, Finegoldia, Blastococcus, Pseudomonas, Bacillus, Micrococcus, Pseudonocardia, Stenotrophomonas, Sanguibacter, Kocuria
N1PCR1 N2PCR1 PCR2	7	Brevundimonas, Variovorax, Rubellimicrobium, Jeotgalicoccus, Alloiococcus, Acidovorax, Carnobacterium
N1PCR1 N2PCR1 PCR3	8	Brachybacterium, Solirubrobacter, Skermanella, Alloprevotella, Frigoribacterium, Prevotella, Ornithinimicrobium, Chryseobacterium
N1PCR1 PCR2 PCR3	8	Streptomyces, Microbacterium, Afipia, Jiangella, Modestobacter, Diaphorobacter, Neisseria, Methylobacterium
N2PCR1 PCR2 PCR3	9	Allorhizobium-Neorhizobium-Pararhizobium-Rhizobium, Bosea, Paenibacillus, Salmonella, Phenylobacterium, Escherichia-Shigella, Pantoea, Acinetobacter, Granulicatella
N1PCR1 N2PCR1	17	Capnocytophaga, hgcI clade, Burkholderia-Caballeronia-Paraburkholderia, Photobacterium, Porphyromonas, Ensifer, Citricoccus, Pseudokineococcus, Lautropia, Oligella, Macrococcus, Pseudoxanthomonas, Gordonia, Ferrovibrio, Shewanella, Dialister, Promicromonospora
N1PCR1 PCR2	2	Mesorhizobium, Aquipuribacter
N1PCR1 PCR3	8	Geodermatophilus, Empedobacter, Rudaea, Cellvibrio, Lysinibacillus, Fusicatenibacter, Cloacibacterium, Flavobacterium
N2PCR1 PCR2	3	Leuconostoc, Aeromonas, Serratia
N2PCR1 PCR3	4	Domibacillus, Ammoniphilus, Quadrisphaera, Microlunatus
PCR2 PCR3	24	Tepidimonas, Anoxybacillus, Propioniciclava, Aerococcus, Pseudarthrobacter, Agrococcus, Varibaculum, Ramlibacter, Clostridium, sensu, stricto, Rheinheimera, Nocardiopsis, Enterobacter, Nonomuraea, Actinotalea, Parvimonas, Gaiella, Saccharopolyspora, Lysobacter, Hyphomicrobium, Mycobacterium, Idiomarina, Curtobacterium, Aquabacterium, Naasia
N2PCR1	9	Pedobacter, Collinsella, Aggregatibacter, Knoellia, Craurococcus, Leifsonia, Dyadobacter, Enhydrobacter, Flavisolibacter
PCR2	51	Altererythrobacter, Undibacterium, Stenotrophobacter, Bifidobacterium, Jatrophihabitans, Candidatus, Udaeobacter, Actinobacillus, Psychromonas, Niveibacterium, Gemmatimonas, Rhodoferax, Nitrospira, Luteolibacter, Bdellovibrio, Limnohabitans, Rhodococcus, Noviherbaspirillum, Turicibacter, Nevskia, [Eubacterium], brachy, group, Lechevalieria, Morganella, Luteitalea, Oxalobacter, [Eubacterium], yurii, group, Ethanoligenens, Amaricoccus, Pseudoclavibacter, Thermaerobacter, Aliterella, CENA595, Marmoricola, Kingella, Thermicanus, Sphingopyxis, S31, Calothrix, PCC-6303, Blautia, Kluyvera, Novibacillus, Opitutus, Veillonella, Weissella, Fimbriiglobus, Rhizobacter, Kurthia, Kineococcus, Tuberibacillus, Prauserella, Tetrasphaera, Roseococcus, Atopobium
PCR3	51	Glutamicibacter, Gemmobacter, Vulcaniibacterium, Bacteroides, Halomonas, Ezakiella, Caulobacter, Adhaeribacter, Ignavigranum, MND1, Faecalibacterium, Alishewanella, Phaselicystis, Aneurinibacillus, Oceanobacillus, Dermabacter, Bergeyella, Porphyrobacter, Luteimonas, Erythrobacter, Actinoplanes, Hymenobacter, Flavihumibacter, Abiotrophia, Sphingoaurantiacus, Pseudorhodoferax, Tatumella, Desulfovibrio, Nakamurella, Rubrobacter, Chryseomicrobium, Microcella, Devosia, Proteiniclasticum, Belnapia, Thermomonas, Leptothrix, Jonesia, Proteiniphilum, Roseburia, Methylocella, Qipengyuania, Conyzicola, metagenome, WCHB1-32, Haliangium, Friedmanniella, Truepera, Lysinimicrobium, Acidibacter, Lachnoanaerobaculum
